# Use of novel structural features to identify urinary biomarkers during acute kidney injury that predict progression to chronic kidney disease

**DOI:** 10.1186/s12882-023-03196-0

**Published:** 2023-06-19

**Authors:** Jennifer R. Charlton, Teng Li, Teresa Wu, Kimberly deRonde, Yanzhe Xu, Edwin J. Baldelomar, Kevin M. Bennett

**Affiliations:** 1 School of Medicine, Department of Pediatrics, Division Nephrology, University of Virginia, Box 800386, Charlottesville, VA 22903 USA; 2 Department of Computer Science, School of Computing and Augmented Intelligence, Arizona State University, Tempe, AZ USA; 3 Department of Industrial Engineering, School of Computing and Augmented Intelligence, Arizona State University, Tempe, AZ USA; 4 Washington University in St. Louis, Mallinckrodt Institute of Radiology, St. Louis MO, USA

**Keywords:** Biomarker, CFE-MRI, Nephron number, Folic acid nephropathy

## Abstract

**Background:**

A significant barrier to biomarker development in the field of acute kidney injury (AKI) is the use of kidney function to identify candidates. Progress in imaging technology makes it possible to detect early structural changes prior to a decline in kidney function. Early identification of those who will advance to chronic kidney disease (CKD) would allow for the initiation of interventions to halt progression. The goal of this study was to use a structural phenotype defined by magnetic resonance imaging and histology to advance biomarker discovery during the transition from AKI to CKD.

**Methods:**

Urine was collected and analyzed from adult C57Bl/6 male mice at four days and 12 weeks after folic acid-induced AKI. Mice were euthanized 12 weeks after AKI and structural metrics were obtained from cationic ferritin-enhanced-MRI (CFE-MRI) and histologic assessment*.* The fraction of proximal tubules, number of atubular glomeruli (ATG), and area of scarring were measured histologically. The correlation between the urinary biomarkers at the AKI or CKD and CFE-MRI derived features was determined, alone or in combination with the histologic features, using principal components.

**Results:**

Using principal components derived from structural features, twelve urinary proteins were identified at the time of AKI that predicted structural changes 12 weeks after injury. The raw and normalized urinary concentrations of IGFBP-3 and TNFRII strongly correlated to the structural findings from histology and CFE-MRI. Urinary fractalkine concentration at the time of CKD correlated with structural findings of CKD.

**Conclusions:**

We have used structural features to identify several candidate urinary proteins that predict whole kidney pathologic features during the transition from AKI to CKD, including IGFBP-3, TNFRII, and fractalkine. In future work, these biomarkers must be corroborated in patient cohorts to determine their suitability to predict CKD after AKI.

**Supplementary Information:**

The online version contains supplementary material available at 10.1186/s12882-023-03196-0.

## New and noteworthy

This study provides a new approach to identify urinary biomarkers. In a mouse model of AKI, we identified several novel urine proteins that predicted a combination of structural changes within the kidney that were observed using cationic ferritin enhanced-magnetic resonance imaging and histologic examination months after injury. This is the first study to develop predictive urinary biomarkers based on imaging metrics of the whole kidney during the transition from acute to chronic kidney disease.

## Introduction

Acute kidney injury (AKI) is common in adults, children, and neonates [1–4]. AKI is caused by pharmacologic or physical insults that lead to varying amounts of damage, and AKI is the leading risk factor for progression to chronic kidney disease (CKD) [5–8]. There is a recognized need to identify patients who will progress to CKD after AKI, in order to apply early interventions to prevent end stage kidney disease. However, it is not currently possible to identify these patients.

Current clinical measurements of the risk of CKD after AKI are based on estimated glomerular filtration rate (GFR) and proteinuria. However, there is significant variability in the decline of GFR between individuals, and an unpredictable rate of decline of GFR in individuals [9]. Many nephrologists believe that “nephron reserve”, or the number of nephrons left after injury has resolved, could better predict which patients with AKI will develop CKD. New technologies that can directly measure nephron number, and other structural changes, may therefore provide better benchmarks for the development of urinary biomarkers following AKI.

A low nephron endowment and nephron loss can reduce the functional capacity of the kidney and worsen the long-term prognosis of patients with AKI [10–12]. In humans, nephrogenesis is complete at the time of full-term birth [13], and no new nephrons can be formed after the progenitor cells differentiate. Therefore, the recovery of kidney function after injury is most likely enabled by compensatory hyperfiltration of the remaining nephrons [14]. Patients with more nephrons can likely maintain GFR after nephron loss compared to those with fewer nephrons. However, nephron number and nephron loss cannot be measured using standard clinical tools.

For decades, there has been strong interest in developing urinary biomarkers to predict the development of CKD after AKI [15, 16], before changes in GFR or proteinuria. Urinary biomarkers such as NGAL, IL-18, KIM-1 and L-FABP have been extensively studied, but only a few have been approved for clinical use. In a 2020 consensus statement [16], three biomarkers were found to have a potential role in clinical prediction of kidney recovery; two from plasma, (hepatocyte growth factor [17, 18] and proenkephalin A [19]), and one from urine, (C–C motif chemokine ligand 14 [20]). Only one urinary biomarker potentially predicted kidney recovery, highlighting an unmet need for sensitive and specific urinary biomarkers to predict which patients with AKI will develop CKD. There is also a longstanding need to validate these candidate biomarkers using pathological structural or functional features associated with CKD.

Several recent advances in imaging techniques have provided measurements of structural features of pathology that may facilitate the discovery of new biomarkers. Cationic ferritin enhanced-MRI (CFE-MRI), in particular, provides a unique, three-dimensional view of the nephron distribution in the entire kidney, allowing nephron number and loss to be mapped in relationship to other anatomical features [21–27]. In CFE-MRI, MRI-detectable cationic ferritin (CF) is injected intravenously, or directly into the renal artery, and binds transiently to the glomerular basement membrane [28, 29], allowing detection and measurement of each glomerulus, in vivo and ex vivo [21–23, 27]. Metrics derived from CFE-MRI can be used in combination with histopathologic examination to comprehensively characterize animal models of AKI and CKD [27]. CFE-MRI is a powerful three dimensional tool and reveals the distribution of pathology throughout the entire kidney. CFE-MRI can be used in longitudinal studies [23], to potentially develop biomarkers of CKD and to determine response to therapies. Importantly, a combination of CFE-MRI and urinary biomarkers could be used to test whether the urinary biomarkers are sensitive to specific structural changes associated with pathology.

The goal of this study was to develop a novel methodology to identify urinary biomarkers that predict the structural changes of in the transition from AKI to CKD. We identified urinary biomarkers at an early and late timepoint in the transition between AKI to CKD using a mouse model of AKI induced by folic acid which recapitulates the pathologic findings of AKI observed in humans [30]. We then measured glomerular number (N_glom_) and apparent volume (aV_glom_) in the kidneys using ex vivo CFE-MRI*,* 12 weeks after injury [27]. We hypothesized that urinary biomarkers at the time of AKI would correlate with permanent structural changes 12 weeks after injury. We further hypothesized that urinary biomarkers 12 weeks after injury will correlate to structural changes in the kidney at that time. We demonstrate that imaging features from CFE-MRI can be used to discover novel biomarkers during the transition from AKI to CKD, and we propose several new candidate urinary biomarkers for further investigation.

## Methods and materials

### Animals

All animal experiments were performed at the University of Virginia and approved by the Institutional Animal Care and Use Committee in accordance with the National Institutes of Health Guide for the Care and Use of Laboratory Animals. This study is reported in accordance with ARRIVE guidelines. The animals used in the present study were a subgroup of a previous study to establish the phenotype of the model [27] (experimental group n = 9, controls, n = 8). There was no power calculation performed for this pilot study. Animals were included in the present study if urine was collected from them at both time points. Adult male C57Bl/6 mice were randomized to receive intraperitoneal folic acid (125 mg/kg body weight in 0.3 M NaHCO_3_, n = 5), and with 10–20 ml/kg of 0.9% NaCl subcutaneously following AKI. Folic acid crystallizes in the tubules, creating an obstruction and inducing AKI and cell death. This model of folic acid-induced AKI recapitulates the major pathologic processes of inflammation, fibrosis, cell death and proliferation in human AKI. The control group received only a single dose of 0.3 M NaHCO_3_ (n = 7) to control for the injection provided in the experimental group. Due to weight loss following AKI, the experimenter could not be blinded to whether the animal received folic acid or not, but blinding was possible at the time of euthanasia. Only male mice were included in this pilot study because the leakage of CF through the glomerulus, typical in female mice, requires a different CFE-MRI protocol that is currently being validated.

**Fig. 1:**
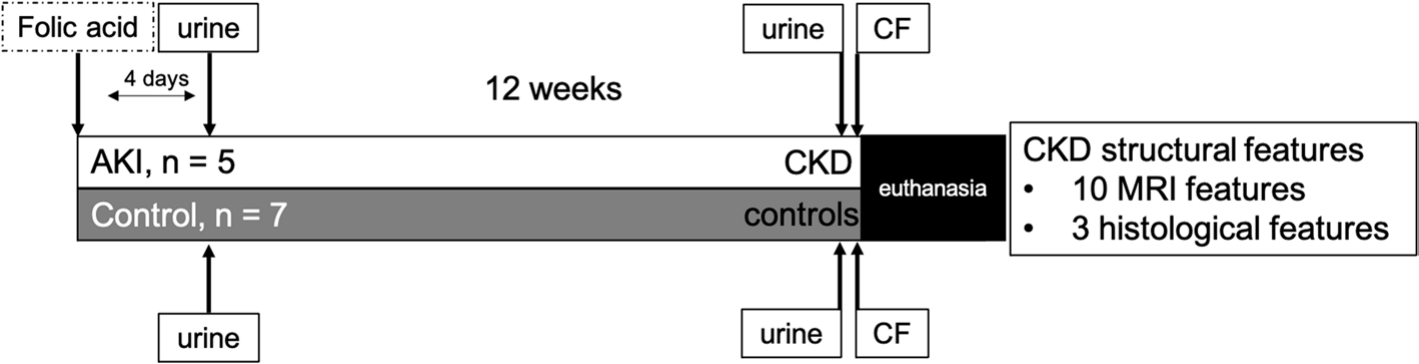
Experimental timeline. Animals were injected with folic acid and urine was collected fours day and 12 weeks after injury. Chronic kidney disease features were assessed 12 weeks after injury and included data from MRI and histologic evaluations

At four days after folic acid (AKI) and at 12 weeks after folic acid (CKD), each animal was placed in an individual plastic container and urine was collected when the animal spontaneously voided. The urine was extracted with a pipette and immediately placed on ice and stored for analysis. Each mouse received horse spleen cationic ferritin (CF: 5.75 mg/100 g body weight, Sigma Aldrich F7879, St. Louis, MO) in two injections, followed by euthanasia with tribromoethanol solution and intracardiac perfusion. The experimental timeline is shown in Fig. 1. The CF-labelled kidneys were imaged ex vivo as previously described [27], and the images were processed using Fiji, Matlab (The Mathworks, Natick, MA), and Python (Python Software Foundation, https://www.python.org/).

### Structural features: CFE-MRI and histology

Structural features derived from CFE-MRI and histology were measured 12 weeks after folic acid induced-AKI. The features derived from CFE-MRI included kidney volume, total number of glomeruli (N_glom_), and mean and median apparent volume of glomeruli (aV_glom_). In those that received folic acid, there were areas where only perfused glomeruli were present, with no surrounding tubules. These areas were termed “cortical lesions”, and the volume, number, and ratio of volume of the lesions to the cortical volume were measured. N_glom_ and aV_glom_ were measured in the glomeruli outside of these lesions. Proximal tubular fraction and % atubular glomeruli were measured on histologic sections stained with Lotus lectin and scarred area was measured using Periodic Acid Schiff and trichrome stained slides as previously published [27]. Details of these methods are included in the Additional file 1 (Supplemental Materials and sFigure 1).

### Urine biomarkers

We measured urinary concentrations of 80 cytokines using the Quantibody Mouse Cytokine Array 4 and Array 5 Kit (RayBiotech, Peachtree Corner, GA). The complete list of target cytokines is shown in Additional file 1 (Supplemental Table 1). These were selected because they include inflammatory and growth factors, each of which are plausible biomarkers. Biomarkers were excluded from further analysis if the urinary concentration from more than two animals in the group was below the threshold of detection. Of the 80 biomarkers, 30 were included for analysis at the AKI timepoint (4 days) and 29 were included for the analysis at the CKD timepoint (12 weeks). Urine creatinine was measured by DetectX Urinary Creatinine Detection Kit (Arbor Assay, K002-H1) [31]. Urinary biomarkers were recorded both as raw measurements and normalized to urine creatinine.

### Data analysis

We evaluated the association between the urinary biomarkers and structural features from CFE-MRI and histology in two ways. First, we determined the correlation between each individual urinary biomarker at either the time of AKI or CKD, and the individual CFE-MRI or histologic features measured at the time of CKD. We did not identify strong correlations with statistical significance, so we further investigated whether a combination of multiple features would be more robust, using principal component analysis (PCA, Python, version 3.9, sklearn package version 1.2.2). PCA is broadly used to compare multivariate datasets [32]. Each principal component (PC) is a linear combination of the original features. We derived PCs from the CFE-MRI and histologic features as structural measures. We then calculated the correlation between the individual urinary biomarkers and the PCs. To derive the PCs, we first subtracted the average of the structural features and scaled them to unit variance between kidneys. We derived two sets of PCs: one set from the MRI and histologic features, and one set from MRI only. We performed separate analyses of the group exposed to folic acid and the control group because the structural features of each group were different. For example, the control group had no scarred areas or cortical lesions, so these metrics were not included in the PCs of the control group. We identified the PCs that covered > 95% variance. Consequently, there were three PCs derived from the CFE-MRI and histologic data, and only two PCs derived from the CFE-MRI data alone. We report the experimental groups (FA = folic acid, control = placebo) and corresponding features (ALL = MRI and histology; MRI = MRI alone) in subscript. For example, PC1_FA_ALL_ was derived from animals who received folic acid and includes features from CFE-MRI and histologic features. The coefficients in each PC were constrained by the sum of squares, and the coefficients spanned [-1,1]. The higher the absolute value, the greater contribution of this structural feature to the corresponding PC. A negative coefficient indicates a negative contribution to the PC. We performed a Pearson’s correlation (MATLAB, corr function) to identify the strength of the correlations. Our sample size was small [33], so we conducted a permutation test (custom program coded in MATLAB, 2021b) to determine the significance of the correlations between the urinary and structural biomarkers and PCs. We report biomarkers with an absolute Pearson’s correlation > 0.85 and a *p*-value < 0.05 in the Results.

## Results

**Fig. 2:**
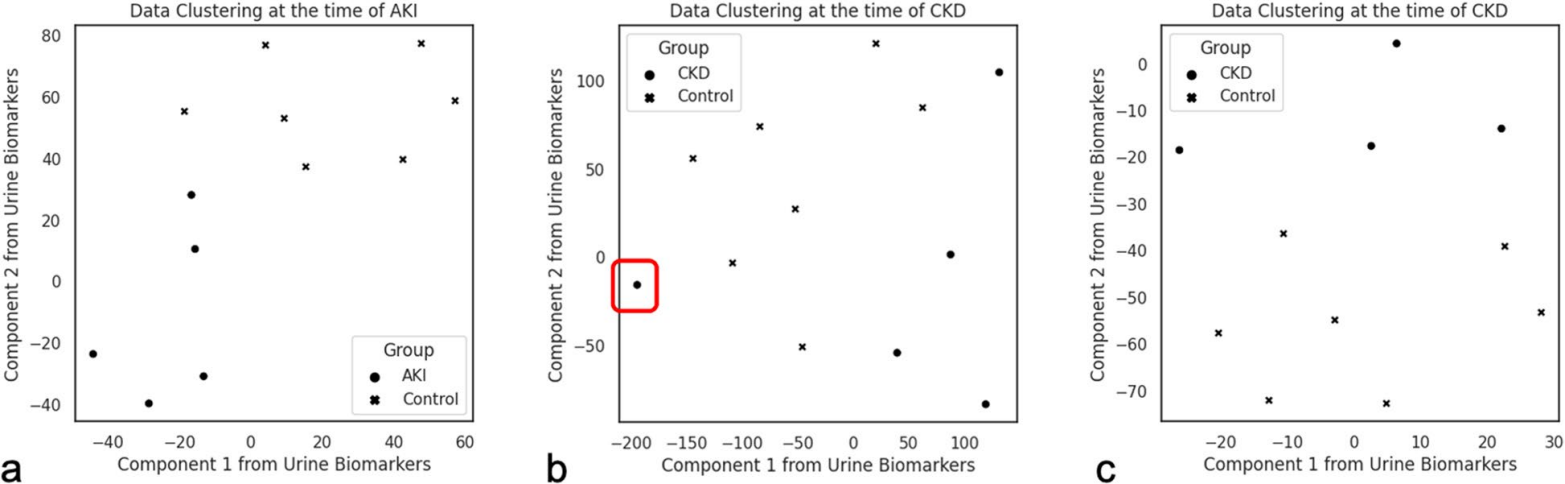
Clustering of urinary biomarkers by t-SNE. The 30 urinary biomarkers at AKI and the 29 urinary biomarkers at CKD were used to generate a t-SNE. The clustering pattern is clear between the folic acid group and controls at the AKI timepoint (**a**). **b** is derived from the urinary biomarker at the CKD timepoint, and contains one outlier (outlined in the red box). If this outlier were removed, the clustering between the folic acid and controls is clear (**c**)

Twelve weeks after exposure to folic acid, the animals had a significantly lower body weight (BW), kidney weight (KW), and KW/BW compared to controls. Glomerular number (N_glom_) was significantly lower in the folic acid group than in controls, but average glomerular volume (aV_glom_) and kidney volume were not significantly different. Histologic metrics such as proximal tubular fraction, atubular glomeruli, and scarred area were different between the groups. These data are summarized in Table 1. A summary of the urinary biomarkers at the time of AKI and CKD is shown in Tables 2 and 3, respectively. We performed t-Distributed Stochastic Neighborhood Embedding (t-SNE), an unsupervised non-linear technique, to visualize the high-dimensional urinary biomarker data (Fig. 2A-C).

**Table 1:** Summary of CKD features: demographic, MRI and histology

	Folic acid (*n* = 5)		Control (*n* = 7)		
	median	IQR	median	IQR	*p* value
Demographic features					
body weight (g)	26.6	24.9–29.2	29.7	29.2–32.5	0.01
total kidney weight (mg)	28	26.0–35.3	43.2	42.4–47.5	< 0.01
kidney weight/body weight (mg/g)	1	1–1.3	1.5	1.3–1.5	0.01
MRI features					
N_glom_	7042	6541–10,743	11,399	10,576–12,079	0.03
mean aV_glom_ (× 10^–4^ mm^3^)	2.71	2.1–3.8	1.9	1.6–2.7	0.14
median aV_glom_ (× 10^–4^ mm^3^)	1.7	1.2–2.1	1.26	1.2–1.7	0.43
kidney volume (mm^3^)	129	115–207	214	191–235	0.07
cluster number	92	11.5–145	0	0–0	< 0.01
cluster volume (mm^3^)	9.6	2.7–13.7	0	0–0	< 0.01
cluster volume/cortical volume (mm^3^/mm^3^)	7.9	1.4–11.4	0	0–0	< 0.01
N_glom_ out of cluster	5970	5548–10,604	11,399	10,368–12,079	0.03
mean aV_glom_ out of cluster (× 10^–4^ mm^3^)	2.4	2.0–3.1	1.9	1.6–2.4	0.14
median aV_glom_ out of cluster (× 10^–4^ mm^3^)	1.7	1.2–2.0	1.3	1.2–1.7	0.53
Histologic features					
scarred (%)	12.5	4.7–19.8	0	0–0	< 0.01
ATG (%)	75.7	66–81	28.9	23.8–33.5	< 0.01
PT area (%)	35	32.5–43	54	51.5–57	< 0.01

*Abbreviations*: *FA* folic acid, *N*_*glom*_ number of glomeruli, *aV*_*glom*_ apparent volume of glomeruli, *ATG* atubular glomeruli, *PT* proximal tubule

**Table 2:** Summary of urinary biomarkers at the time of AKI

	Folic acid (*n* = 5)		Control (*n* = 7)		
	median	IQR	median	IQR	*p* value
Urinary Biomarkers					
AR	0.00	0–0	0.74	0–21.96	0.24
Axl	303.06	150.90–515.89	1659.98	49.13–3226.85	0.02
CD40	345.16	152.00–446.65	19.39	0–24.22	< 0.01
CXCL16	144.07	83.93–284.91	14.68	0.16–31.12	< 0.01
EGF	10,201.58	4881.40–11,647.78	6501.24	3598.84–6920.87	0.16
E-selectin	1541.09	902.50–2521.18	3039.9	1627.47–6907.81	0.03
Fractalkine	133.59	112.88–217.96	743.33	31.03–2812.74	0.06
GITR	30.31	1.31–69.75	86.05	59.14–182.19	< 0.01
HGF	370.64	115.40–1032.81	413.66	213.37–647.03	0.93
IGFBP-2	588.56	330.52–1776.79	1774.91	38.11–3395.89	0.08
IGFBP-3	11,745.16	4678.90–15,510.52	3230.34	13.17–4761.83	0.02
IGFBP-5	1177.33	413.01–2429.06	153.29	37.77–594.62	0.04
IGFBP-6	190.09	36.92–1203.95	10.36	0–60.12	0.15
IGF-1	20.96	4.10–223.73	9.4	2.26–32.28	0.31
IL-1ra	112.61	73.70–203.67	98.25	6.05–892.69	0.62
MDC	2.52	0–100.63	0	0–1.83	0.30
MIP-2	0.00	0–5.11	0.53	0–9.66	0.68
MIP-3a	4.58	1.81–5.80	1.52	0–11.58	0.48
OPN	10,747.71	9398.67–17,027.91	11,155.56	276.5–16,676.27	0.44
OPG	1301.17	183.84–2211.51	0	0–411.07	0.06
Pro-MMP-9	92.58	8.39–183.25	11.42	0–152.67	0.35
P-selectin	3393.98	1321.19–4189.93	4263.04	2580.02–6616.41	0.07
Resistin	33.31	2.98–105.77	19.98	9.46–46.39	0.25
SCF	143.72	27.23–161.04	14.83	4.29–72.08	0.04
SDF-1a	30.88	7.68–144.25	156.05	20.32–626.56	0.08
VCAM-1	8860.70	2871.60–10,859.42	3496.84	374.84–6187.89	0.10
VEGF	211.57	154.27–1088.01	859.55	398.28–2300.37	0.07
bFGF	0.00	0–2.70	19.06	0–30.6	0.03
CD30L	0.00	0–0.005	0.43	0–0.85	0.02
Eotaxin	0.97	0–3.88	5.53	2.85–46.63	0.10
G-CSF	156.73	61.50–726.01	39.17	0–130.62	0.14
ICAM-1	3473.06	500.08–5009.52	4659.89	0–8207.42	0.53
IL-1a	121.63	0.68–798.95	4.04	0.58–615.78	0.46
IL-3	0.36	0–6.69	1.81	0–44.45	0.44
IL-7	0.00	0–32.81	8.36	3.48–14.01	0.79
IL-13	0.00	0–67.90	8.74	0–23.32	0.61
KC	19.61	0–26.79	0.91	0–23.36	0.20
Leptin	1.97	0–6.70	3.16	0–19.58	0.30
MCP-5	0.00	0–20.48	0	0–95.21	0.13
MIP-1a	0.00	0–5.35	14.07	0–36.47	0.02
MIP-1 g	2242.00	457.26–3725.27	263.65	141.53–486.69	0.05
RANTES	3.42	0–79.29	11.27	0.41–120.62	0.74
TARC	5.53	0–9.78	6.24	0–20.36	0.38
TCA-3	0.00	0–53.51	0	0–35.46	0.94
TNF RI	3549.51	1700.84–4042.87	642.62	342.09–1112.21	< 0.01
TNF RII	25,168.29	5020.84–26,561.14	10,398.57	5599.4–16,529.79	0.23

**Table 3:** Summary of urinary biomarkers at the time of CKD

	Folic acid (*n* = 5)		Control (*n* = 7)		
	median	IQR	median	IQR	*p* value
Urinary Biomarkers					
AR	0	0–0	0	0–0.66	0.36
Axl	428.6	220.43–856.42	1258.94	496.32–1503.76	< 0.01
CD40	2.35	0–26.17	1.59	0–9.79	0.45
CXCL16	1.54	0–13.06	4.1	0–6.5	0.82
EGF	6045.65	4249.97–6684.19	6403.45	5788.01–6693.07	0.36
E-selectin	1903.27	1351.13–2918.73	2949.64	1595.48–3856.95	0.07
Fractalkine	479.32	123.74–742.85	1682.09	427.58–1974.74	0.01
GITR	83.94	31.8–104.24	115.28	75.21–126.4	0.07
HGF	239.48	54.97–324.22	238.71	153.01–274.84	0.74
IGFBP-2	2131.79	784.58–4129.26	4498.31	1487.01–5424.8	0.12
IGFBP-3	3348.41	2281.66–3432.74	2653.19	2155.26–3138.09	0.06
IGFBP-5	120.16	110.4–212.08	272.02	98.42–605.47	0.05
IGFBP-6	0	0–37.56	11.27	0–46.23	0.79
IGF-1	56.79	4.33–407.19	11.11	3.7–22.65	0.16
IL-1ra	47.56	19.83–3862.36	656.15	122.97–907.61	0.8
MDC	0	0–0.26	0	0–0	0.37
MIP-2	0.16	0–2.08	0	0–0.4	0.22
MIP-3a	1.16	0.88–21.64	0.46	0–10.82	0.35
OPN	11,167.65	9713.6–14,532.93	15,499.71	9670.07–18,105.13	0.13
OPG	36.1	0–43.82	10.14	0–565.23	0.33
Pro-MMP-9	7.02	0–161.54	0	0–8.26	0.26
P-selectin	1973.84	1591.03–3999.49	2922.07	2459.1–4174.05	0.21
Resistin	29.55	8.55–58.44	23.69	16.22–32.75	0.63
SCF	3.02	0–26.79	6.4	2.72–13.91	0.86
SDF-1a	137.77	26.82–237.62	651.05	37.5–1179.12	0.02
VCAM-1	3429.93	2332.37–3896.82	3639.4	2170.46–5803.86	0.35
VEGF	654.35	396.33–1275.48	919.17	443.13–1471.97	0.52
bFGF	9.51	0–45.56	17.14	0–30.33	0.83
CD30L	0.06	0–0.84	0.15	0–0.51	0.69
Eotaxin	14.75	1.37–39.62	7.84	4.04–16.12	0.35
G-CSF	29.72	7.17–51.46	35.27	19.88–69.85	0.29
ICAM-1	2130.85	825.84–4662.25	6126.93	3553.11–7547.55	< 0.01
IL-1a	0.08	0–93.99	1	0–12.66	0.44
IL-3	0	0–3.32	0.38	0–1.81	0.99
IL-7	0.91	0–8.37	5.08	0–14.7	0.27
IL-13	5.15	0–127.57	31.19	0–76	0.93
KC	0	0–6.76	0	0–10.64	0.94
Leptin	0	0–0	0.17	0–4.42	0.21
MCP-5	0	0–0	0	0–53.5	0.36
MIP-1a	0	0–7.6	2.67	0–32.17	0.16
MIP-1 g	266.57	161.87–579.57	374.07	93.9–422.95	0.85
RANTES	14.18	2.75–16.21	18.79	10.86–24.98	0.08
TARC	0	0–6.21	0	0–27.26	0.37
TCA-3	7.04	0–35.6	16.03	0–47.32	0.51
TNF RI	550.45	530.68–983.43	453	278.37–555.25	0.07
TNF RII	9064.16	4667.71–13,615.03	13,784.38	3899.81–17,151.92	0.39

### AKI urinary biomarkers, and CFE-MRI and histologic features of CKD

**Fig. 3:**
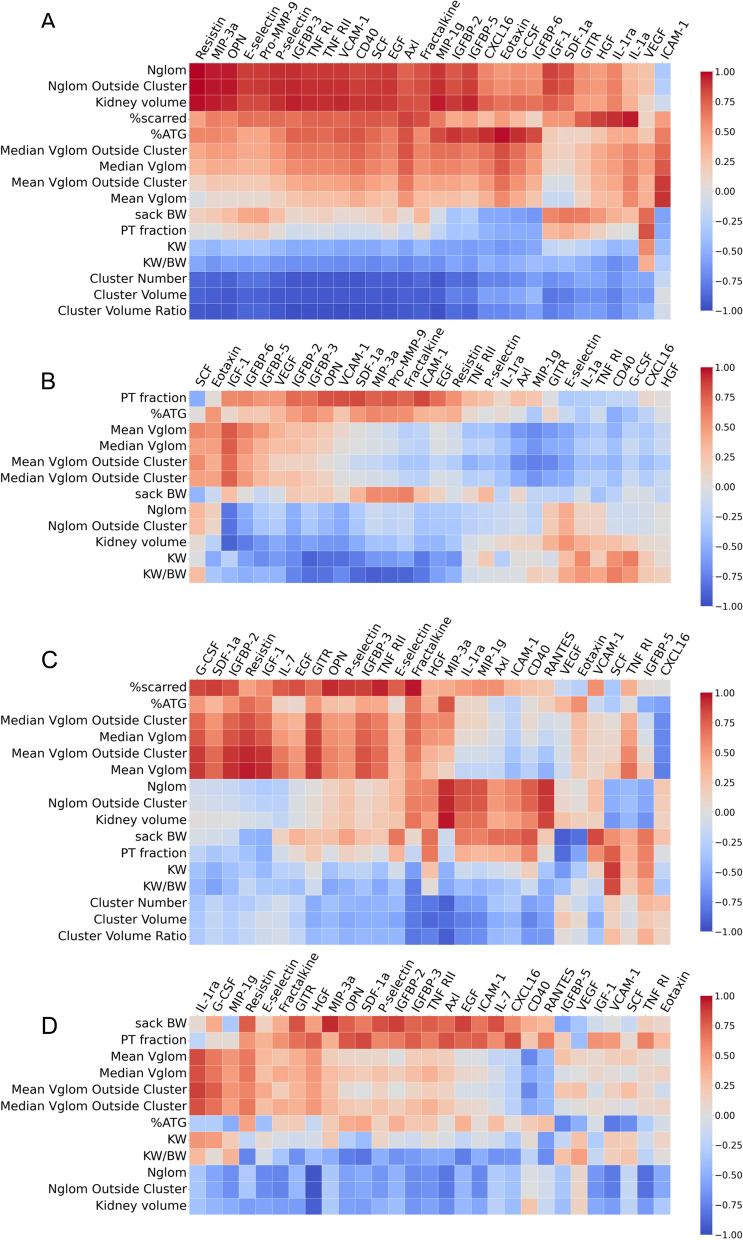
Heatmap of the correlation between urinary biomarkers at the time of AKI and CFE-MRI and histologic features at the time of CKD: folic acid (**a**) and controls (**b**). Heatmap of the correlation between individual urinary biomarkers and CFE-MRI and histologic features at the time of CKD: folic acid (**c**) and controls (**d**)

We derived principal components (PCs) from MRI and histologic data. In animals that received folic acid, three PCs were derived from 13 features including 10 from CFE-MRI and three from histology (PC_FA_ALL_). The subscript for each PC begins with the experimental group (folic acid (FA) vs control) followed by the features included to derived the PC (ALL-histologic, MRI vs MRI- MRI alone). In the control group, three PCs (PC_control_ALL_) were derived from 12 features including seven from CFE-MRI and two from histologic measures. Figure 3A and B summarize the correlation between individual urinary biomarkers at the time of AKI, and CFE-MRI and histologic features at the time of CKD. Table 4 summarizes the coefficients of the structural features indicating the contribution of each to the PC and sFigures 2 and 3 demonstrate the variance from each feature in the PC.

**Table 4:** Principal components derived from MRI and histologic features

	PC1 components		PC2 components		PC3 components	
Variables included to derive PC	FA	control	FA	control	FA	control
N_glom_	-0.25	-0.34	-0.39	0.38	-0.05	0.09
mean aV_glom_ (× 10^–4^ mm^3^)	-0.21	0.39	0.45	0.26	0.11	-0.08
median aV_glom_ (× 10^–4^ mm^3^)	-0.3	0.37	0.29	0.32	0.11	0.01
kidney volume (mm^3^)	-0.28	-0.39	-0.31	0.25	-0.24	-0.15
cluster number	0.34	NA	0.12	NA	-0.13	NA
cluster volume (mm^3^)	0.32	NA	0.17	NA	-0.23	NA
cluster volume/cortical volume (mm^3^/mm^3^)	0.33	NA	0.17	NA	-0.05	NA
N_glom_ outside clusters	-0.27	-0.35	-0.37	0.37	-0.07	0.07
mean aV_glom_ outside clusters (× 10^–4^ mm^3^)	-0.25	0.37	0.39	0.29	0.06	-0.18
median aV_glom_ outside cluster (× 10^–4^ mm^3^)	-0.31	0.37	0.24	0.33	0.1	-0.01
scarred area (%)	-0.26	NA	0.13	NA	-0.17	NA
ATG (%)	-0.32	-0.02	0.13	0.32	-0.19	0.83
PT area (%)	0.04	0.21	-0.16	-0.44	0.87	0.48

*Abbreviations*: *FA* folic acid, *N*_*glom*_ number of glomeruli, *aV*_*glom*_ apparent volume of glomeruli, *ATG* atubular glomeruli, *PT* proximal tubule

Fourteen urinary biomarkers were significantly correlated with PC1_FA_ALL_ or PC1_control_ALL_ (Table 5, *p* < 0.05). Twelve were detected in the AKI group (Axl, CD40, EGF, IGFBP-3, IGFBP-5, Pro-MMP-9, P-selectin, SCF, VCAM-1, MIP-1 g, TNF RI, and TNF RII) and two were detected in the control group (IGFBP-6 and IGF-1). Urinary VEGF correlated with PC3_FA_ALL_. Proximal tubular fraction provided the greatest contribution to PC3_FA_ALL_. The average urinary IGFBP-3 in the AKI group was 10,299 pg/ml (range: 4679–15,510) and in the control group was 3,074 pg/ml (range: 13–4762). The average urinary TNF RII in the AKI group was 17,481 pg/ml (range: 5020–26,561), and in the control group was 10,212 pg/ml (range: 5599–16,529). These data indicate that there are several biomarkers at the time of AKI that predict the CKD phenotype, but only urinary VEGF appears to predict tubular damage (PC3_FA_ALL_).

**Table 5:** Correlation between PCs and raw or normalized urinary biomarkers at the time of AKI

**PC**	**Urinary biomarker**	**Raw**	**Normalized**^a^
Folic acid			
PC1_FA_ALL_	Axl	-0.68 (0.08)	-0.96 (0.0005)
	CD40	-0.86 (0.03)	-0.96 (0.0014)
	EGF	-0.89 (0.03)	-0.89 (0.01)
	*IGFBP-3*	*-0.91 (0.02)*	*-0.92 (0.007)*
	IGFBP-5	-0.44 (0.22)	-0.86 (0.007)
	Pro-MMP-9	-0.88 (0.03)	-0.76 (0.05)
	P-selectin	-0.85 (0.04)	-0.85 (0.02)
	SCF	-0.85 (0.04)	-0.92 (0.01)
	VCAM-1	-0.88 (0.03)	-0.94 (0.005)
	MIP-1 g	-0.89 (0.03)	-0.94 (0.005)
	TNF RI	-0.86 (0.04)	-0.93 (0.007)
	*TNF RII*	*-0.94 (0.008)*	*-0.92 (0.01)*
PC2 _FA_ALL_	ICAM-1	0.83 (0.05)	0.88 (0.03)
PC3 _FA_ALL_	VEGF	0.86 (0.04)	0.90 (0.03)
Control			
PC1 _control_ALL_	IGFBP-6	0.94 (0)	0.69 (0.05)
	IGF-1	0.71 (0.04)	0.94 (0.002)
PC2 _control_ALL_	Eotaxin	0.89 (0.01)	0.47 (0.15)
PC3 _control_ALL_	MIP-3a	0.91 (0.003)	0.89 (0.004)
	Pro-MMP-9	0.89 (0.005)	0.89 (0.003)

Pearson correlation (permutation *p* value)

Italics: Both raw and normalized had a Pearson’s correlation > 0.90

^a^normalized to urine creatinine (mg/dl)

### CKD urinary biomarkers, and CFE-MRI and histologic features of CKD

Both raw and normalized concentrations of fractalkine correlated significantly with PC1_FA_ALL_ (r^2^ of -0.93 for raw concentration and -0.86 for the normalized concentration). The mean concentration of urinary fractalkine was 449 pg/ml (range: 124–743) in the CKD group and was 1299 pg/ml (range: 428–1974) in controls. Fractalkine was the only urinary biomarker that correlated with PC1_FA_ALL_, but both SCF and VEGF correlated with PC3_FA_ALL_ (Table 6). Both raw and normalized concentrations of SCF strongly correlated with PC3_FA_ALL_ (raw: 0.89, normalized: 0.89), but normalized urinary VEGF was inversely correlated with PC3_FA_ALL_ (normalized: -0.87). These data indicate that 12 weeks after injury, urinary fractalkine strongly correlates with the CKD phenotype, whereas urinary VEGF and SCF appear consistent with tubular damage (PC3). The correlation between the individual urinary biomarkers and CFE-MRI and histologic features 12 weeks later is summarized in a heatmap in Fig. 3C and D.

**Table 6:** Correlation between PCs and raw or normalized urinary biomarkers at the time of CKD

Folic acid			
**PC**	**Urinary biomarker**	**Raw**	**Normalized**^a^
PC1 _FA_ALL_	Fractalkine	-0.93 (0.008)	-0.86 (0.02)
PC2 _FA_ALL_	IGF-1	0.73 (0.09)	0.88 (0.03)
	Resistin	0.59 (0.17)	0.85 (0.04)
	MIP-1 g	-0.90 (0.01)	-0.64 (0.12)
	RANTES	-0.89 (0.009)	-0.88 (0.01)
PC3 _FA_ALL_	SCF	0.89 (0.02)	0.89 (0.02)
	VEGF	-0.69 (0.08)	-0.87 (0.01)

Pearson correlation (permutation *p* value)

^a^normalized to urine creatinine (mg/dl)

### AKI and CKD urinary biomarkers derived from only CFE-MRI features

We created an additional set of PCs to determine if urinary biomarkers could predict the MRI features alone. In both the AKI and CKD groups, two PCs were derived from 10 CFE-MRI features in the folic acid group (PC1_FA_MRI_ & PC2_FA_MRI_) and from seven CFE-MRI features in the control group (PC1_control_MRI_ and PC2_control_MRI_). The contributions of each of the individual components are shown in Table 7.

**Table 7:** Principal component derived from only MRI features

	PC1 components		PC2 components	
Variables included to derive PC	Folic acid	control	Folic acid	control
N_glom_	-0.3	-0.32	-0.36	0.52
mean aV_glom_ (× 10^–4^ mm^3^)	-0.2	0.41	0.49	0.25
median aV_glom_ (× 10^–4^ mm^3^)	-0.3	0.4	0.35	0.3
kidney volume (mm^3^)	-0.32	-0.37	-0.29	0.36
cluster number	0.38	NA	0.06	NA
cluster volume (mm^3^)	0.37	NA	0.11	NA
cluster volume/cortical volume (mm^3^/mm^3^)	0.37	NA	0.12	NA
N_glom_ outside clusters	-0.31	-0.33	-0.34	0.52
mean aV_glom_ outside clusters (× 10^–4^ mm^3^)	-0.25	0.4	0.44	0.29
median aV_glom_ outside cluster (× 10^–4^ mm^3^)	-0.33	0.4	0.3	0.31

Eleven urinary biomarkers were strongly correlated with PC1; nine were derived from the folic acid group (PC1_FA_MRI_: Axl, CD40, EGF, IGFBP-3, SCF, VCAM-1, MIP-1 g, TNF RI and TNF RII) and two from the control group (PC1_control_MRI_: IGFBP-6 and IGF-1). Eotaxin correlated with PC2_control_MRI_ in the control group. Table 8 shows raw and normalized data.

**Table 8:** Correlation between PCs and raw or normalized urinary biomarkers at the time of AKI using only MRI features

Folic acid			
**PC**	**Urinary biomarker**	**Raw**	**Normalized**^a^
PC1_FA_MRI_	Axl	-0.6 (0.14)	-0.95 (0.002)
	CD40	-0.8 (0.046)	-0.97 (0.0008)
	EGF	-0.84 (0.04)	-0.91 (0.01)
	IGFBP-3	-0.87 (0.03)	-0.94 (0.003)
	IGFBP-5	-0.41 (0.25)	-0.87 (0.02)
	MIP-3a	-0.57 (0.15)	-0.87 (0.02)
	OPN	-0.81 (0.04)	-0.86 (0.02)
	Pro-MMP-9	-0.88 (0.03)	-0.80 (0.04)
	P-selectin	-0.83 (0.04)	-0.88 (0.02)
	SCF	-0.79 (0.05)	-0.93 (0.008)
	VCAM-1	-0.83 (0.04)	-0.95 (0.003)
	MIP-1 g	-0.85 (0.03)	-0.95 (0.003)
	TNF RI	-0.80 (0.049)	-0.94 (0.003)
	*TNF RII*	*-0.90 (0.02)*	*-0.94 (0.006)*
Control			
		**Raw**	**Normalized**^a^
PC1_control_MRI_	IGFBP-6	0.92 (0.002)	0.66 (0.07)
	IGF-1	0.75 (0.04)	0.92 (0.004)
PC2_control_MRI_	Eotaxin	0.92 (0.01)	0.45 (0.16)

Pearson correlation (permutation *p* value)

Italics: Both raw and normalized had a Pearson’s correlation > 0.90

^a^normalized to urine creatinine (mg/dl)

At the time of CKD, urinary fractalkine was strongly correlated with PC1_FA_MRI_ (Table 9). Both raw and normalized values of fractalkine significantly inversely correlated with PC_FA_MRI_ (R^2^ of -0.92 for raw urine and -0.83 for the normalized urine). RANTES inversely correlated well with PC2_FA_MRI_ in the folic acid group (R^2^ for raw: -0.93, normalized: -0.87). The mean raw concentration in urine was 11.3 pg/ml (range: 2.8–16.2) in the CKD group and 17.8 pg/ml (range: 10.9–25.0) in the control group. In the control group, the raw urinary resistin correlated with PC2_control_MRI_, but the normalized concentration did not (r^2^: raw 0.84, normalized: 0.14). The data indicate that urinary fractalkine could predict a phenotype of CKD that was measured by multiple features derived from CFE-MRI.

**Table 9:** Correlation between PCs and raw or normalized urinary biomarkers at the time of CKD using only MRI features

Folic acid			
** PC**	**Urinary biomarker**	**Raw**	**Normalized**^a^
PC1 _FA_MRI_	fractalkine	-0.92 (0.01)	-0.83 (0.04)
PC2 _FA_MRI_	MIP-1 g	-0.86 (0.02)	-0.58 (0.13)
PC2 _FA_MRI_	RANTES	-0.93 (0.005)	-0.87 (0.01)
Control			
		**Raw**	**Normalized**^a^
PC2 _control_MRI_	resistin	0.84 (0.02)	0.14 (0.36)

Pearson correlation (permutation *p* value)

^a^normalized to urine creatinine (mg/dl)

## Discussion

This study demonstrates the feasibility of using structural features measured from CFE-MRI and histology to develop urinary biomarkers that predict early phase CKD. This study is unique because it incorporates nephron-level measurements of pathology in the whole kidney with structural features to provide a multi-feature phenotype of CKD, allowing identification of urinary biomarkers that were acquired at the time of AKI and that predict the phenotype. This approach should allow for sensitive identification of candidate predictive biomarkers, which can then be tested in a clinical study.

We identified urinary biomarkers at the time of AKI and CKD that are highly correlated to the structural pathology of CKD, and several urinary biomarkers at the time of AKI predicted CKD in the kidney 12 weeks after injury. At the time of AKI, urinary IGFBP-3 and TNF RII strongly correlated with the structural features of CKD. Twelve weeks after AKI, urinary fractalkine correlated strongly with structural features of CKD. This study supports the use of novel structural features, measured from MRI and histology, to identify urinary biomarkers in the transition from AKI to CKD.

Most clinical studies have relied on functional metrics such as GFR to validate urinary biomarkers [16]. These functional metrics are primarily sensitive to late-stage progression of CKD. To our knowledge, there has not been a study to develop urinary biomarkers using whole-kidney metrics associated with progressive structural pathologies, like those measured using CFE-MRI. Several urinary biomarkers, both at the time of CKD and 12 weeks earlier at the time of AKI, were associated with the structural changes associated with CKD in the tubule and glomerulus. We propose these urinary biomarkers for further evaluation in clinical studies.

At the time of AKI, urinary IGFBP-3 and TNF RII had the strongest correlation to the structural changes of CKD. Although the IGF pathway, including IGF-1, IGF-2 and the binding proteins, is important in AKI [34], there is little known about the direct role of IGFBP-3 in AKI progression. IGFBP-3 is mainly expressed in the female reproductive system and less in the kidney, but binds a large majority of circulating IGF-1 [35, 36]. IGFBP-3 is important in a variety of kidney diseases. IGFBP-3 confers a pro-apoptotic effect in diabetic nephropathy. In patients with FSGS, urinary excretion of IGFBP-3 correlates with disease activity, and urinary levels of IGFBP-3 appear to distinguish FSGS from minimal change disease [37]. TNF RII has been more extensively studied as a biomarker of AKI. The Biological Markers of Recovery for the Kidney (BioMaRK) study showed that plasma concentrations of TNF RII along with IL-6, IL-18, IL-10, and TNF RI were inversely correlated with kidney recovery [38]. TNF RII concentration in the urine was not reported in the BioMaRK study.

In some cases, it may not be known if a patient has had AKI, and urinary biomarkers could be used to determine if there is chronic damage, prior to a GFR decline. Here, we showed urinary fractalkine was highly associated with the structural phenotype of CKD. Fractalkine is a chemokine, also known as CX3CL1, that recruits innate immune cells into the kidney [39]. Systemic activation of the fractalkine axis may play a role in CKD-associated cardiovascular disease [40]. After injury, mesangial, endothelial and tubular cells all have increased expression of fractalkine [41, 42]. In 2010, Koziolek and colleagues showed a strong correlation between the expression of fractalkine and fibrosis in a study of female CD-1 mice exposed to folic acid [42]. Fractalkine was detected in the endothelial cells and proximal tubular cells by immunohistochemical techniques at late phases after injury (142 days after injury). Other studies have also shown that the expression of fractalkine in the kidney appears to occur later in the progression from AKI to CKD [43, 44].

This study has a few limitations. We present urinary biomarker data normalized to urine creatinine to adjust for variability in the concentrations of the samples. However, secretion of creatinine significantly contributes to the final urine creatinine concentration in mice. Recognizing this limitation, we reported both raw and normalized urinary biomarker values. The model of injury with folic acid is an obstructive AKI and is similar to kidney injury from medications such as acyclovir. The histologic findings after folic acid-induced AKI are very similar to human pathology [30]. This established model results in a variable CKD phenotype, similar to AKI in humans. It is possible that biomarkers observed in the urine are not specific to the kidney. This concern is somewhat mitigated by prior observations of the specificity of several of these biomarkers to kidney injury in animal models and humans [37, 38, 42–44]. However, further work is required to validate any proposed biomarker for human use. Further work is needed to determine whether urinary biomarkers can be used to predict earlier pathologic changes to more precisely guide and monitor new therapies in patients after AKI.

AKI is a complex syndrome with a variety of factors that lead to a decline in renal function. There is a great need to develop noninvasive biomarkers to stratify patients for therapeutic studies based on risk of developing CKD, and to guide clinical care based on this risk. This study demonstrates that structural features derived from both MRI and histology can be used to generate noninvasive urinary biomarkers at the time of AKI to predict the progression of kidney disease.

## Supplementary Information

**Additional file 1: sFigure 1.** MRI and histologic evaluation of kidneys. **sFigure 2.** Boxplot of normalized MRI and histologic features of folic acid mice (*n*=5). **sFigure 3.** Boxplot of normalized MRI and histologic features of control mice (*n*=7). **sTable 1.** Potential urinary biomarkers from cytokine array Q4&Q5.

## Data Availability

The datasets used and/or analyzed during the current study are available from the corresponding author on reasonable request.
