# Supplementary material for: Use of novel structural features to identify urinary biomarkers during acute kidney injury that predict progression to chronic kidney disease
Source: BMC Nephrol. 2023 Jun 19;24:178. doi: 10.1186/s12882-023-03196-0 (PMC10278294; doi:10.1186/s12882-023-03196-0)
Supplement: Supplementary file 1 — Additional file 1: sFigure 1. MRI and histologic evaluation of kidneys. sFigure 2. Boxplot of normalized MRI and histologic features of folic acid mice (n=5). sFigure 3. Boxplot of normalized MRI and histologic features of control mice (n=7). sTable 1. Potential urinary biomarkers from cytokine array Q4&Q5. [file 12882_2023_3196_MOESM1_ESM.docx]

**Supplemental Materials:**

1. Supplemental Methods
   1. Proximal tubule (PT) fraction
   2. Atubular glomeruli
   3. Scarred area
2. Supplemental Figures
   1. sFigure 1. MRI and histologic evaluation of kidneys.
   2. sFigure 2. Boxplot of normalized MRI and histologic features of folic acid mice (n=5).
   3. sFigure 3. Boxplot of normalized MRI and histologic features of control mice (n=7).
3. Supplemental Tables
   1. sTable 1: Potential urinary biomarkers from cytokine array Q4&Q5
4. **Supplemental Methods**
5. **Proximal tubule (PT) fraction and atubular glomeruli**

In mid-coronal kidney sections, *Lotus tetragonolobus* lectin (Vector Laboratories) was applied to identify the proximal tubules. Lotus lectin binding is specific to the mature proximal tubular cells and papillary collecting duct. The sections were treated with proteinase K followed by biotinylated *Lotus* lectin (1:50 dilution) and induction of the ABC-DAB reaction. The DAB reaction was analyzed in the cortex of each image to quantify the area of the proximal tubules using the Amira software (FEI, Bordeaux, France). Using a stereological approach^31-33^, ten fields under the capsule were photographed at 20x magnification. The DAB reaction product was expressed as a percent area value (volume fraction [V_v_]).

1. **Atubular glomeruli**

To establish the number of atubular glomeruli, the mid-coronal kidney section stained with Lotus was imaged using the Grundium Ocus slide scanner. In mice, the lack of lotus lectin staining in Bowman’s capsule is either a disruption of the glomerulotubular (GT) connection, an atubular glomerulus or a section of the glomerulus that does not contain the GT connection. Validation of this method in serial sections has been previously published^34^. We report the number of lotus+ glomeruli over the total number of glomeruli in the section.

1. **Scarred area**

Mid-coronal sections were stained using the Masson’s trichrome stain performed in the Research Histology Core laboratory at the University of Virginia. Images were imported into Amira software, where the cortex and medulla were manually segmented. Wedge shaped areas in the subcapsular region, where there was collagen deposition and no tubules, were segmented from the rest of the cortex. The % of scarred area was calculated by area of collagen deposition/total cortical area.

1. **Supplemental Figures**

**sFigure 1. MRI and histologic evaluation of kidneys.** Two dimensional representative slices from 3D Gradient-echo MR images of control (a) and folic acid (b) kidney. The green circles outline the areas of cortical lesions where glomeruli clustered with no tubules. The trichrome stain was used to highlight areas of fibrosis and this was reported a %scarring (c and d). Lotus staining was used to highlight the proximal tubules and to discern the connection between the glomerulus and the tubule. The healthy controls have more area occupied by proximal tubules (e) than those exposed to folic acid (f). Highlighted in a yellow circle is a glomerulus without lotus+ staining. Scale bar=100 microns.


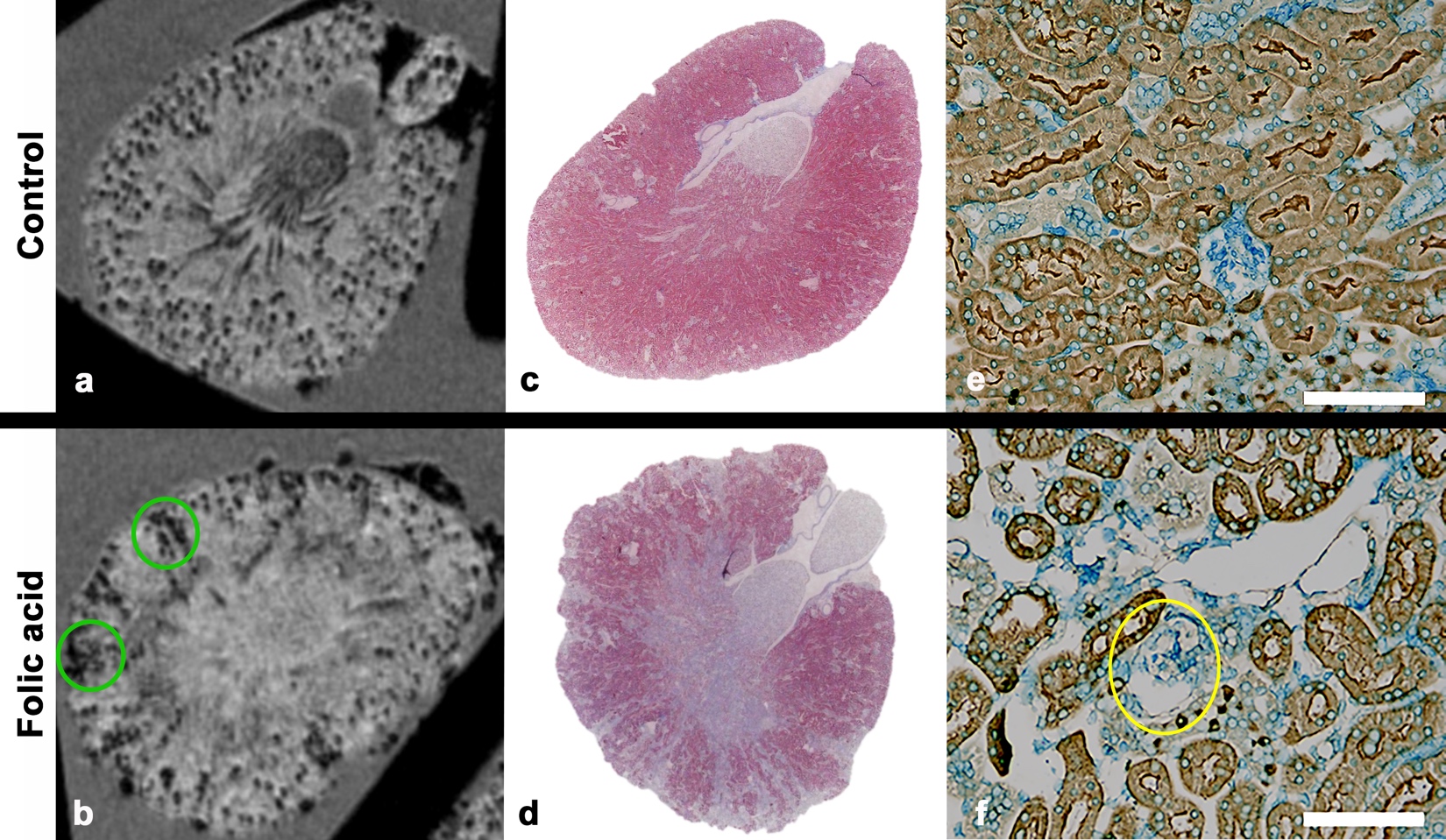


**sFigure 2.** Boxplot of normalized MRI and histologic features of folic acid mice (n=5).

**
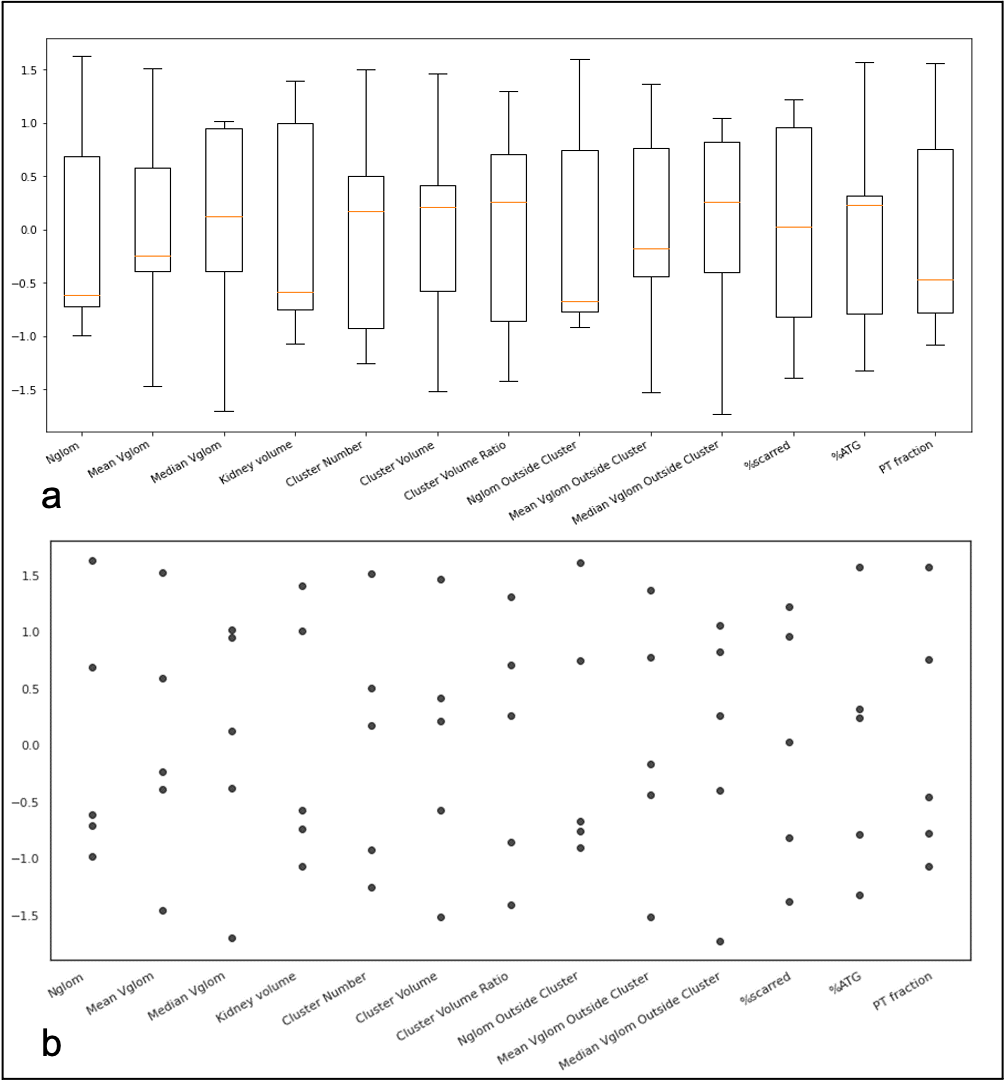
**

**sFigure 3.** Boxplot of normalized MRI and histologic features of control mice (n=7).


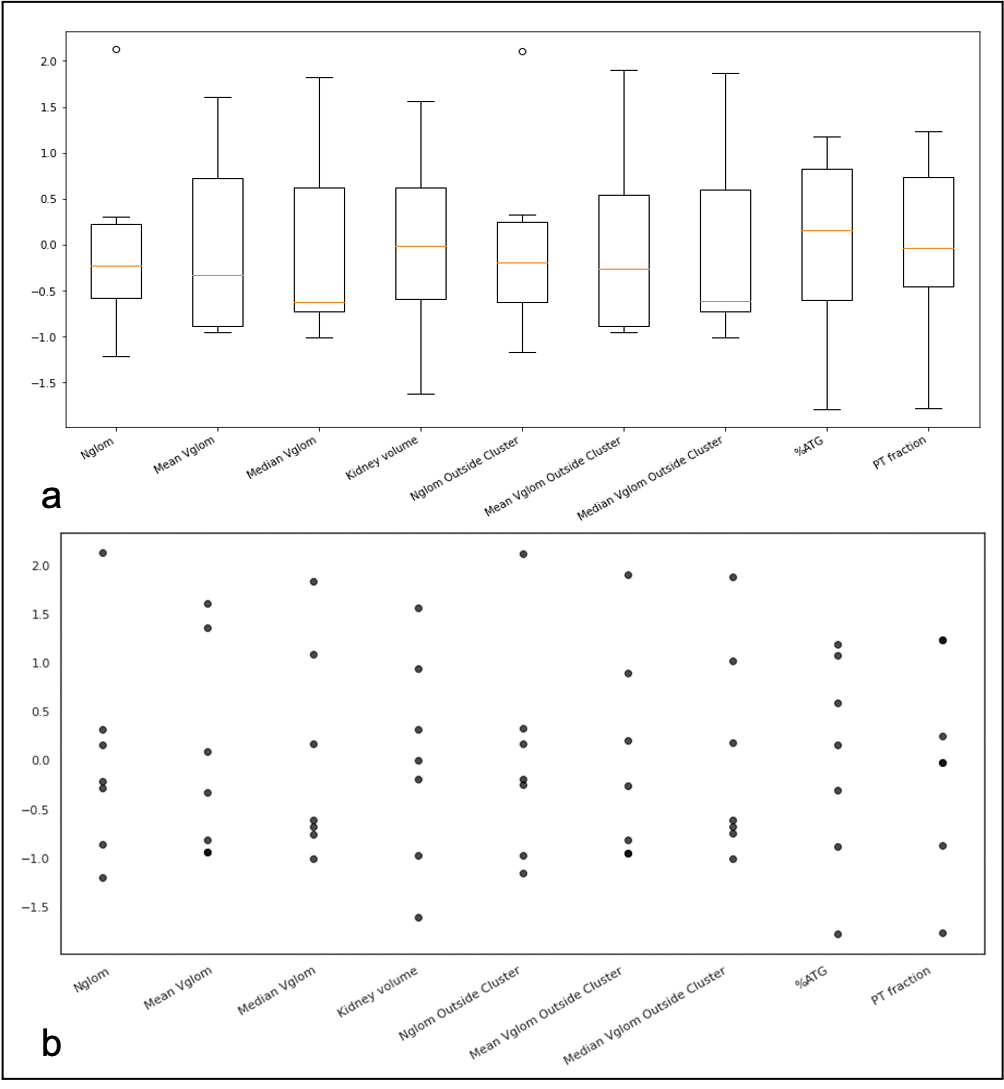


1. **Supplemental Table**

**sTable 1: Potential urinary biomarkers from cytokine array Q4&Q5**

| ***Amphiregulin*** | Axl | CD27 Ligand (TNFSF7) | CD30 (TNFRSF8) | CD40 (TNFRSF5) |
| --- | --- | --- | --- | --- |
| CXCL16 | EGF | E-Selectin | Fractalkine (CX3CL1) | GITR (TNFRSF18) |
| HGF | IGF-1 | IGFBP-2 | IGFBP-3 | IGFBP-5 |
| *IGFBP-6* | IL-1 ra (IL-1 F3) | IL-12 p70 | IL-17E (IL-25) | IL-17F |
| IL-2 R alpha | IL-20 | IL-23 p19 | IL-28A (IFN-lambda 2) | I-TAC (CXCL11) |
| ***MDC (CCL22)*** | ***MIP-2*** | MIP-3 alpha (CCL20) | Osteopontin (SPP1) | ***Osteoprotegerin (TNFRSF11B)*** |
| Prolactin | *Pro-MMP-9* | P-Selectin | Resistin | SCF |
| SDF-1 alpha (CXCL12 alpha) | Thrombopoietin (TPO) | VCAM-1 (CD106) | VEGF-A | VEGF-D |

| ***bFGF*** | **BLC (CXCL13)** | ***CD30 Ligand*** ***(TNFSF8)*** | **Eotaxin-1 (CCL11)** | **Eotaxin-2 (MPIF-2/CCL24)** |
| --- | --- | --- | --- | --- |
| **Fas Ligand (TNFSF6)** | **GCSF** | **GM-CSF** | **ICAM-1 (CD54)** | **IFN-gamma** |
| ***IL-1 alpha* *(IL-1 F1)*** | **IL-1 beta (IL-1 F2)** | **IL-2** | ***IL-3*** | **IL-4** |
| **IL-5** | **IL-6** | **IL-7** | **IL-10** | **IL-12 p40** |
| ***IL-13*** | **IL-15** | **IL-17A** | **IL-21** | ***KC*** ***(CXCL1)*** |
| ***Leptin*** | **LIX** | **MCP-1 (CCL2)** | ***MCP-5*** | **M-CSF** |
| **MIG (CXCL9)** | ***MIP-1 alpha*** ***(CCL3)*** | **MIP-1 gamma** | **Platelet Factor 4 (CXCL4)** | **RANTES** **(CCL5)** |
| ***TARC*** ***(CCL17)*** | ***I-309*** ***(TCA-3/CCL1)*** | **TNF alpha** | **TNF RI (TNFRSF1A)** | **TNF RII (TNFRSF1B)** |

Urinary biomarkers in bold were removed at the AKI timepoint and those in italics were removed at the CKD timepoint.
